# The predictive value of methylene blue dye as a single technique in breast cancer sentinel node biopsy: a study from Dharmais Cancer Hospital

**DOI:** 10.1186/s12957-017-1113-8

**Published:** 2017-02-07

**Authors:** Bayu Brahma, Rizky Ifandriani Putri, Ramadhan Karsono, Bob Andinata, Walta Gautama, Lenny Sari, Samuel J. Haryono

**Affiliations:** 1 Department of Surgical Oncology, Dharmais Cancer Hospital, Jalan. Let. Jend. S. Parman Kav. 84-86, Jakarta, 11420 Indonesia; 2 Department of Anatomical Pathology, Dharmais Cancer Hospital, Jalan. Let. Jend. S. Parman Kav. 84-86, Jakarta, 11420, Indonesia; 3 Department of Surgical Oncology, Bogor City General Hospital, Jalan DR. Semeru No. 120, West Bogor, West Java 16112 Indonesia; 4 Departement of Surgical Oncology, Mochtar Riady Comprehensive Cancer Center (MRCCC) Siloam Hospital, Jalan Garnisun Dalam No. 2-3, Semanggi, Central Jakarta, 12930 Indonesia

**Keywords:** Sentinel node, Breast cancer, Axillary lymph node, Methylene blue dye

## Abstract

**Background:**

Axillary lymph node dissection (ALND) has been the standard treatment of breast cancer axillary staging in Indonesia. The limited facilities of radioisotope tracer and isosulfan or patent blue dye (PBD) have been the major obstacles to perform sentinel node biopsy (SNB) in our country. We studied the application of 1% methylene blue dye (MBD) alone for SNB to overcome the problem.

**Methods:**

This prospective study enrolled 108 patients with suspicious malignant lesions or breast cancer stages I–III. SNB was performed using 2–5 cc of 1% MBD and proceeded with ALND. The histopathology results of sentinel nodes (SNs) were compared with axillary lymph nodes (ALNs) for diagnostic value assessments.

**Results:**

There were 96 patients with invasive carcinoma from July 2012 to September 2014 who were included in the final analysis. The median age was 50 (25–69) years, and the median pathological tumor size was 3 cm (1–10). Identification rate of SNs was 91.7%, and the median number of the identified SNs was 2 (1–8). Sentinel node metastasis was found in 53.4% cases and 89.4% of them were macrometastases. The negative predictive value (NPV) of SNs to predict axillary metastasis was 90% (95% CI, 81–99%). There were no anaphylactic reactions, but we found 2 cases with skin necrosis.

**Conclusions:**

The application of 1% MBD as a single technique in breast cancer SNB has favorable identification rates and predictive values. It can be used for axillary staging, but nevertheless the technique should be applied with attention to the tumor size and grade to avoid false negative results.

## Background

Breast cancer is the most common malignancy, accounting for 31.2% of all cancers and 26.5% as the cause of cancer death among women in our hospital [1]. In Asia-Pacific region, 12% of breast cancer incidence rates and 17% of its death occur in Indonesia [2]. The information of axillary lymph node (ALN) metastasis is one of the most important prognostic factors in breast cancer treatments. It is conventionally determined by axillary lymph node dissection (ALND) [3, 4]. This procedure gives morbidities such as lymphedema, loss of sensory, limited mobility, and seroma formation which will decrease the quality of life [4–6]. Nowadays, breast cancer treatments have moved towards conservation therapies, and sentinel node biopsy (SNB) has been introduced as a part of the minimal invasive breast surgery [3, 7]. Unfortunately, ALND is still the standard procedure for axillary staging in Indonesia. The limitation to provide sophisticated technologies for SNB has been our mainstay issue.

The work by Morton et al. [8] in cutaneous melanoma was the turning point of the acceptance of the sentinel node (SN) concept. It was soon adopted to breast cancer patients by using isosulfan blue dye or radioisotope tracer alone to find SNs. Initially, the reported identification rates of SNs ranged between 65 and 98% and false negative rates between 0 and 5% [9–12]. In developed countries, the optimal technology for SNB uses isosulfan or patent blue dye (PBD), preoperative lymphoscintigraphy, and radioisotope tracer, which are used as a single or combination technique [9–13]. As an alternative to these devices, several studies have been conducted to validate 1% methylene blue dye (MBD) for SNB. Simmons [14] was the first surgeon who reported the successful application of 1% MBD in breast cancer SNB. The other studies also supported its use because of the favorable results in identification and false negative rates, fewer allergic complications, and lower cost [15–22].

Limited access to PBD and radioisotope tracer is the main problem to perform SNB in Indonesia. Not to mention our geographic distribution of the population, the availability and cost to provide nuclear medicines, or gamma probes in every hospital have contributed to the difficulty for administering SNB. Recently, we have started to use 1% MBD alone, and the initial results from 24 patients were favorable with the identification rates of 95.8% [23]. As we have moved towards better breast cancer care, it is important for us to conduct a study to overcome the limitation to perform SNB. The primary objective of the study is to evaluate the identification rates and negative predictive value (NPV) of SNs to predict axillary metastasis by using 1% MBD alone.

## Methods

### Participants

In this study, 108 consecutive patients with diagnosis of breast cancer or suspicious malignancy were enrolled prospectively at Dharmais Cancer Hospital, Bogor City General Hospital, and Mochtar Riady Comprehensive Cancer Center (MRCCC) Siloam Hospital between July 2012 and September 2014. There were five surgeons participating in the research. SJH, RK, and WG had more than 5 years of experience, while BB and BA had more than 3 years of experience in breast cancer surgery including ALND. All surgeons had less than 10 cases in performing MBD technique alone prior to the study. BB, RK, and SJH were also the surgeons who were working and undertaking SNB in the other participating hospitals besides Dharmais Cancer Hospital. We included patients with any tumor size (T) without palpable ALNs (cNo) and had performed core needle or fine needle aspiration (FNA) biopsy. Patients without final pathological results of invasive breast cancer or had a pregnancy were excluded from the study. The Institutional Review Board at Dharmais Cancer Hospital approved the study, and all patients were provided informed consent.

### Sentinel node biopsy and axillary lymph node dissection

SNB was performed using 1% MBD. It was injected in a subareolar or peritumoral area with the dose of 2 until 5 cc. We did a peritumoral injection in all cases with previous excisional biopsy at the upper outer quadrant of the breast or according to the surgeon’s preferences. A breast massage was done for 5 min after the injection. In a standard breast conserving or oncoplastic breast conserving surgery (BCS), a separate incision in the lower axillary hairline was made to find SNs before lumpectomy or quadrantectomy. When the patients underwent mastectomy, SNB was undertaken through the same mastectomy incision before removing the breast. Sentinel nodes were defined as blue nodes or lymph nodes with a lymphatic blue channel. All procedures proceeded to ALND levels I–II. Axillary lymph node dissection level III was done when there were suspicious lymph node metastases at level II. If a frozen section were available, it would be used to assess an intraoperative SN metastasis. Histopathological results of all ALNs were collected after the surgery.

### Pathological examination

The sentinel nodes were surgically removed at the beginning of the surgical procedure and sent for a standard pathological assessment or frozen-section examination if available. The sentinel nodes were sectioned no thicker than 2 mm and parallel to the long axis. An intraoperative analysis was categorized to positive or negative for metastases. The rest of SNs were formalin fixed and paraffin sectioned with hematoxyline-eosin staining.

The tumors were histologically classified according to the World Health Organization (WHO) Histological Classification of Breast Tumors, and grading was defined according to Elston and Ellis modification [24]. All specimens were reviewed in Dharmais Cancer Hospital by two pathologists (RIP and LS). Only 2 patients who underwent surgery at MRCCC hospital were not reviewed due to the patients’ preference and thus analyzed by using the original histopathology report from the local pathologist. Molecular subtypes for invasive cancer were classified as luminal A (ER+ and/or PR+, HER2−, and histological grade either 1 or 2), luminal B (ER+ and/or PR+, HER2+; or ER+ and/or PR+, HER2− and grade 3), triple negative (ER−, PR−, HER2−), and HER2+ (ER−, PR−, HER2+) [25].

The nodal involvement was classified according to the 6th edition of the American Joint Committee on Cancer (AJCC) manual. Macrometastasis (MAC) is defined as tumor deposits larger than 2 mm, micrometastasis (MIC) if tumor deposits between 0.2 and 2.0 mm, and isolated tumor cells (ITCs) if there are cell clusters or a single cell no larger than 0.2 mm. Serial sections and immunohistochemistry (IHC) for cytokeratin were performed when there was some doubt to define ITCs. The rest of ALNs were also examined in a similar manner. The histopathology of SNs was compared to the final examination of ALNs for the presence of metastases [24, 26].

### Statistical analysis

Descriptive data were presented in the table of frequency. Sensitivity (Se), specificity (Sp), positive predictive value (PPV), and negative predictive value (NPV) were calculated using CATmaker. Diagnostic values were reported with 95% confidence of interval (CI). We used SPSS version 16.0 to manage the data.

## Results

### Patient characteristics

We prospectively enrolled 108 patients from July 2012 to September 2014. Twelve patients with FNA biopsy result of suspicious breast cancer were excluded because the frozen section and final pathological results were not invasive carcinoma. There were 87 (90.6%) patients from Dharmais Cancer Hospital and 9 (9.4%) from the other hospitals. Of the 96 patients who were included in the final analysis, the median age was 50 years (range 25–69 years). There were 9 (9.4%) patients in stage I, 64 (66.7%) in stage II, and 23 (23.9%) in stage III. The median pathological tumor size was 3 (1–10) cm. Invasive carcinoma of no special type (NST) was the most common result which accounted for 71 (74%) patients and invasive lobular carcinoma (ILC) in 11 (11.5%) patients. We classified breast cancer molecular profile based on IHC examination. Thirty-eight (39.6%) patients were classified as luminal A breast cancer, 24 (25%) as luminal B, 10 (10.4%) as HER2+ type, and 24 (25%) as triple negative (TNBC). Mastectomy was the most common surgical procedure which was done in 60 (62.5%), meanwhile BCS in 36 (37.5%) patients. Table 1 summarizes the characteristic of patients.

**Table 1:** Patient characteristics (*n* = 96)

Patient characteristics		Number	Percentage
Age (years)	Median (range)	96	50 (25–69)
Tumor size	Median (range)	96	3 (1–10)
Pathology	NST	71	74.0
	ILC	11	11.4
	Others	14	14.6
Molecular subtypes	Luminal A	38	39.6
	Luminal B	24	25.0
	HER2 positive	10	10.4
	Triple negative	24	25.0
Surgery	Mastectomy	60	62.5
	BCS	36	37.5

*NST* no special type, *ILC* invasive lobular carcinoma, *BCS* breast conservation surgery

### Sentinel node biopsy and pathological examination

**Fig. 1:**
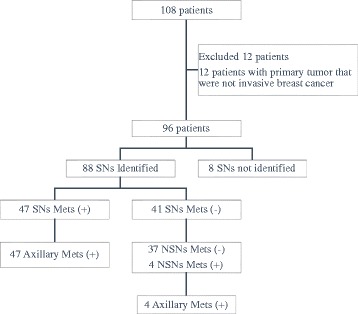
Patients flowchart for recruitment and SN assessment to predict axillary metastasis. *SNs* sentinel nodes, *NSNs* non-sentinel nodes, *Mets* metastasis

We could identify SNs in 88 patients. Therefore, the SNs identification rate was 91.7%. Peritumoral injections were done in 29 (30.2%) and subareolar in 67 (69.8%) cases. The median number of SNs that could be identified was 2 (1–8) and the median of ALNs was 11 (5–27). In this group where SNs were identified, the number of SNs without metastases was 41. Four of these patients were found to have metastases in non-sentinel nodes (NSNs), and so the total patients without lymph node metastases were 37 (42%). There were 47 (53.4%) cases with SN metastases and 42 (89.4%) of them had MAC. The number of SN metastases which was only found in 1–2 SNs was 43 (91.5%), whereas 4 (8.5%) metastases were identified in more than 2 SNs. We discovered 25 (53.2%) cases with additional metastatic deposits in NSNs. Therefore, in 22 (46.8%) patients, the metastases only occurred in SNs. Table 2 describes the cases with positive SNs. The SNs detected metastases in 47 of 51 cases, resulting in a Se of 92% (95% CI, 85–100%), and there were 4 NSN metastases in the SN negative group which resulted in a NPV of 90% (95% CI, 81–99%). All 4 cases that failed to predict ALN metastases had a median pathological tumor size of 4 cm, 2 patients in stage IIB and the others were in stage IIIA. Three (75%) patients were grade 3 invasive carcinoma. Figure 1 and Table 3 show the recruitment of patients and results of diagnostic value. Table 4 describes the false negative patients

**Table 2:** Sentinel node characteristics of patients with positive metastases (*n* = 47)

SN characteristic		Number	Percentage
Positive SN count	1–2	43	91.5
	>2	4	8.5
Metastasis type	Macrometastases	42	89.4
	Micrometastases	5	10.6
Patients with SNs only metastasis count		22	46.8
Patients with SN and NSN metastasis count		25	53.2

*SNs* sentinel nodes, *NSNs* non-sentinel nodes

**Table 3:** Diagnostic value of sentinel nodes (*n* = 88)

Se	Sp	PPV	NPV
92% 95% CI (85–100)	100% 95% CI (100–100)	100% 95% CI (100–100)	90% 95% CI (81–99)

*Se* sensitivity, *Sp* specificity, *PPV* positive predictive value, *NPV* negative predictive value, *CI* confidence interval

**Table 4:** Characteristics of patients with false negative SN (*n* = 4)

Patient characteristics		Number	Percentage
Age (years)	Median (range)	4	44 (35–59)
Tumor size	Median (range)	4	4.0 (3.0–6.0)
Pathology	NST	4	100.0
Molecular subtypes	Luminal B	3	75.0
	Triple negative	1	25.0
Tumor grade	2	1	25.0
	3	3	75.0

*NST* no special type

### Unidentified sentinel nodes

The SNs could not be found in 8 patients. The median age of the patients was 54 years old (range 36–67 years) with the median tumor size of 2.8 (1.5–5.0) cm. There were 2 (25%) grade 1, 3 (37.5%) grade 2, and 3 (37.5%) grade 3 invasive carcinoma. Two (25%) patients had lymph node metastases and the rest were negative. Table 5 describes the characteristic of the unidentified SN group.

**Table 5:** Characteristics of patients with unidentified SN (*n* = 8)

Patient characteristics		Number	Percentage
Age (years)	Median (range)	8	54 (36–67)
Tumor size	Median (range)	8	2.8 (1.5–5.0)
Pathology	NST	7	87.5
	Ca with medullary feature	1	12.5
Molecular subtypes	Luminal A	4	50.0
	HER2 positive	2	25.0
	Triple negative	2	25.0
Tumor grade	1	2	25.0
	2	3	37.5
	3	3	37.5
Metastases in LN	Positive	2	25.0
	Negative	6	75.0

*NST* no special type, *Ca* carcinoma, *LN* lymph node

### Complications

Two patients experienced skin necrosis around the injection site after 5 cc of peritumoral injection. They were mastectomies cases one of whom had a breast reconstruction. These patients successfully underwent conservative wound treatment. We found no systemic anaphylactic reactions among all patients.

## Discussion

The paradigm of early breast cancer management has changed toward conservation treatments, and SNB has replaced ALND in terms of axillary staging [27, 28]. In comparison with developed countries, the majority of breast cancer cases in our country are in locally advanced stages [29]. This is the reason why ALND has become a common practice among our surgeons. Nowadays, we have been expecting to treat patients in early stages since the improvement in our national health care insurance and this condition will motivate us to promote SNB. Although the standard for lymphatic mapping supports the combination technique [13, 30], limited access to radioisotope tracers, PBD, and nuclear medicine facilities have become our obstacles. Our population is distributed across islands and not every hospital has sophisticated technologies for SNB. Therefore, we try to overcome this problem by applying 1% MBD alone for SN identification.

The issue of PBD limitation was solved by several authors with the utilization of 1% MBD which had favorable results [17, 21, 31]. The identification rate of 92% from our research was acceptable when it was compared with the other studies that used MBD [14–17, 19–21]. Another research which supported our result was confirmed by Liu et al. in their randomized controlled study in cutaneous melanoma. They found that MBD was as effective as isosulfan blue dye to identify SNs [32]. The median SN number, which was 2 nodes from our study, was equal with the studies that suggested to find 2 until 3 SNs to minimize the false negative rate [33–37].

In the identified SN group, 42% of the cases were lymph node negative for metastases. It means that there were many cases which were not supposed to receive ALND and we could have saved a lot of patients from having the risk of lymphedema and other morbidities. We believe if our surgeons can apply this SNB technique instead of routine ALND, we will make a better quality of life after the surgery and overall reduce the cost of breast cancer treatment in Indonesia and its associated surgical morbidities.

The next important findings from our study were the facts that 53% of metastatic foci were found in SNs and nearly half (47%) of them were only confined in SNs. The early publications of SNB in breast cancer have also reported that approximately 50% patients with SN metastases did not have positive NSNs [38, 39]. In this case, the utility of a nomogram to predict NSN metastases [40–43] would become a valuable tool for us.

The Z0011, IBCSG 23-01, and AMAROS studies have given new perspectives to omit ALND after positive SNs [44–46]. According to the studies, patients with a small-sized tumor, plans for BCS, and whole breast radiation are the suitable indications. These selection criteria did not match with the majority of our patient characteristics because it had been shown in this study that we had bigger median tumor size, 24% cases were in stage III, 89% MAC in SNs, and mastectomy was more common than BCS. As we had 91% patients with 1 until 2 metastases in SNs, the POSNOC trial is expected to give us a better evidence for omitting ALND after positive SNs [47], particularly in mastectomy which represents the majority of our cases.

The reported NPV in this study was 90%, and a randomized study from Canavese et al. nearly had the same result (91.1%) [48]. We realized that our NPV was lower than the other studies (92.3 and 96.1%) [49, 50]. It might have been due to the 4 false negative cases which had bigger median tumor size (4 cm) and higher tumor grade (75% in grade 3). So, there were possibilities that tumor size more than 3 cm and high grade tumors had higher risks of volume nodal metastases and blockage of the lymphatic system to SNs and alternates to false SNs [48, 51]. However, when we analyzed separately by excluding stage III patients (data not shown), the NPV would be 95% (95% CI, 80–100%). Therefore, surgeons must be cautious when performing SNB with MBD alone in a patient with grade 3 and more than 3 cm tumor size. Under these circumstances, looking for additional non blue suspicious lymph nodes is suggested to minimize false negative result.

In this study, SNs could not be found in 8 patients. There are some related factors with the failures to find SNs. The age, body mass index (BMI), tumor size, location, grade, type of previous biopsy, SNB technique, and surgeon’s experience have been reported in literatures as the factors that influence SN identification [52–54]. The median age of the unidentified SN group was 54 years and this older condition could have been one of the factors which accounted for the unsuccessful identification in the final result. The increased fatty tissue in the breast among older patients may decrease lymphatic flow and failures to identify SNs [52, 53].

The surgeon’s experience is another important factor for localizing SNs, especially if blue dye alone is used as the method of choice. Some literatures have explained that identification of SNs will be reduced by less experienced surgeons and the use of blue dye alone technique [53, 54]. Our failure to find SNs might have been explained by these factors as well because in this study, the application of MBD alone was a relatively new technique for us and we did not have many experiences regarding this technique prior to the study.

Higher tumor grade has been known as a negative factor for SN identification in univariate analysis [53]. In our result, grades 2 and 3 tumors constituted about 75% of the cases. Although tumor grade has not been proven as an independent factor for the failure [53], we think it could have contributed to the negative finding in our study.

We experienced two skin necroses around the injection site. Local skin irritation or necrosis after MBD injection was reported by other authors [22, 55]. The toxic effects are due to the formation of aldehydes and a reduction in oxidation products which initiate inflammatory reactions [56]. Although we were not really sure if the skin necrosis was caused by MBD or skin flap necrosis after mastectomy, we decided to lower the dose of injection until 2 cc and we did not have skin necrosis thereafter. We did not find anaphylactic reactions in our cases. The incidence of allergic reactions following PBD was between 0.06 and 2.7% [56]. Whereas anaphylactic reactions following MBD injection was very rare, there were several related serious effects after intrauterine injection [57–60] and pulmonary edema had also been reported after breast cancer SNB in two series [61, 62]. Although MBD can be used safely for lymphatic mapping because of its very rare effects in allergic reactions, we suggest that the operating team should be aware and prepared for the potential anaphylactic reactions of MBD that could happen.

This study had several limitations. First, we only included clinically node negative patients but we did not perform ALN biopsy if the axillary ultrasound found suspicious lymph nodes. Ultrasound-guided axillary lymph node biopsy will select patients with true negative lymph nodes before surgery. Second, blue nodes or non-blue nodes with lymphatic blue channels were the only criteria for SNs. We did not try to find the non-blue suspicious nodes as SNs. These could have reduced our NPV results, especially in cases with high grade and bigger tumor size that could have alternated MBD into the false SNs.

## Conclusions

This study has proven that SNB in breast cancer can be performed with 1% MBD alone. It can be done in clinical settings with limited access to perform the standard combination technique or when PBD is not available. The important factors that should be considered are the following: first, in high grade and bigger tumor size, surgeons must not be satisfied when they only find the blue nodes. The non-blue suspicious lymph nodes must be searched in order to reduce false negative results. Second, a better understanding of the SN anatomic location in the axilla is the key point to increase the identification rate when applying MBD alone technique.

## Abbreviations

ALND: Axillary lymph node
MBD: Methylene blue dye
SNB: Sentinel node biopsy
